# The effects of hemodialysis on the functional brain connectivity in patients with end-stage renal disease with functional near-infrared spectroscopy

**DOI:** 10.1038/s41598-023-32696-0

**Published:** 2023-04-07

**Authors:** Kang Min Park, Chang Min Heo, Dong Ah Lee, Yoo Jin Lee, Sihyung Park, Yang Wook Kim, Bong Soo Park

**Affiliations:** 1 Department of Neurology, Inje University College of Medicine, Busan, Korea; 2 Department of Internal Medicine, Haeundae Paik Hospital, Inje University College of Medicine, Haeundae-ro 875, Haeundae-gu, Busan, Korea

**Keywords:** Neuroscience, Nephrology, Neurology

## Abstract

This study aimed to investigate functional brain connectivity in patients with end-stage renal disease (ESRD) undergoing hemodialysis using functional near-infrared spectroscopy (fNIRS) and to analyze the effect of hemodialysis on functional brain connectivity. We prospectively enrolled patients with ESRD undergoing hemodialysis for > 6 months without any history of neurological or psychiatric disorders. fNIRS data were acquired using a NIRSIT Lite device. Measurements were performed thrice in the resting state for each patient: before the start of hemodialysis (pre-HD), 1 h after the start of hemodialysis (mid-HD), and after the end of hemodialysis (post-HD). We processed and exported all data, and created a weighted connectivity matrix using Pearson correlation analysis. We obtained functional connectivity measures from the connectivity matrix by applying a graph theoretical analysis. We then compared differences in functional connectivity measures according to hemodialysis status in patients with ESRD. We included 34 patients with ESRD. There were significant changes in the mean clustering coefficient, transitivity, and assortative coefficient between the pre- and post-HD periods (0.353 vs. 0.399, *p* = 0.047; 0.523 vs. 0.600, *p* = 0.042; and 0.043 vs. − 0.012, *p* = 0.044, respectively). However, there were no changes in the mean clustering coefficient, transitivity, and assortative coefficient between the pre- and mid-HD periods, or between the mid- and post-HD periods. In addition, there were no significant differences in the average strength, global efficiency, and local efficiency among the pre-, mid-, and post-HD periods. We demonstrated a significant effect of hemodialysis on functional brain connectivity in patients with ESRD. Functional brain connectivity changes more efficiently during hemodialysis.

## Introduction

Chronic kidney disease (CKD) is defined as abnormalities of kidney function for > 3 months^1^. CKD is one of the most common diseases in adults, with an estimated global prevalence of 9.1%^2^. It is divided into five stages according to the glomerular filtration rate (GFR), among which the last stage with a GFR of < 15 mL/min per 1.73 m^2^ is called end-stage renal disease (ESRD)^3^. The treatment of choice in patients with ESRD is renal replacement therapy, which comprises hemodialysis, peritoneal dialysis, and kidney transplantation^4^. Among these, hemodialysis is the dominant form of renal replacement therapy and accounts for 80–90% of all dialysis; however, it varies among countries^4^.

Furthermore, several neurological abnormalities, including cognitive decline, are commonly observed in patients with ESRD^5^. Recently, several research studies have been conducted using neuroimaging to identify brain connectivity changes in patients with ESRD, which are associated with various neurological symptoms such as cognitive decline^6–9^. We have previously explored changes in structural connectivity in patients with ESRD using diffusion tensor imaging and analyzed changes in functional connectivity using resting-state functional magnetic resonance imaging (rs-fMRI)^6^. As a result, there were significant structural and functional connectivity changes confirmed in patients with ESRD compared with the healthy normal group^6^. However, these changes differed depending on the type of renal replacement therapy. Patients undergoing hemodialysis have reported more severe changes in functional brain connectivity, whereas those undergoing peritoneal dialysis have shown more alterations in structural brain connectivity^7^. A recent study using rs-fMRI found very interesting results, showing that some of the brain networks returned to normal after kidney transplantation, including the dorsal attention, central executive, auditory, and visual networks^8,9^. However, to the best of our knowledge, no study has been conducted on the effect of hemodialysis on brain connectivity or networks in patients with ESRD.

Functional brain connectivity is defined as functional connectivity across brain regions dependent on neuronal oscillations. Various modalities can explore functional brain connectivity, each with its strengths and weaknesses. The most commonly used modality to determine functional connectivity is rs-fMRI^6,7^; however, there are also other various methods, including electroencephalography (EEG)^10^, magnetic encephalography^11^, and positron emission tomography, to investigate functional connectivity^12^. One modality recently used to determine functional connectivity is functional near-infrared spectroscopy (fNIRS)^13^. fNIRS is an optical technique that uses near-infrared spectroscopy to monitor the brain^14,15^. Brain activity is measured using near-infrared light to estimate cortical hemodynamic activity in response to neural activity. fNIRS is a noninvasive technique that can be utilized in mobile settings. It is generally known to be more convenient to use, and has superior temporal resolution compared with rs-fMRI and better spatial resolution than EEG^14,15^.

In this study, we investigated functional brain connectivity in patients with ESRD undergoing hemodialysis using fNIRS and analyzed the effect of hemodialysis on functional brain connectivity. To achieve this, fNIRS was conducted thrice consecutively in the same patient: before, during, and after hemodialysis. We hypothesized that functional brain connectivity would change more efficiently during hemodialysis in patients with ESRD.

## Methods

### Participants: patients with ESRD

This prospective study was approved by the Institutional Review Board (IRB) of Haeundae Paik Hospital, and all methods were performed in accordance with the guidelines and regulations (IRB number: HPIRB 2022-6-004-003). All patients were informed of the research process, and informed consent was obtained before the study. We enrolled patients with ESRD between June 2022 and September 2022 at our hospital based on the following criteria: (1) clinically diagnosed ESRD, with a GFR < 15 ml/min/1.73 m^2^, requiring renal replacement therapy^1,3^, (2) received hemodialysis for > 6 months, and (3) no history of neurological or psychiatric disorders.

Hemodialysis-related components such as dialyzer use, blood flow, and dialysate flow rate were controlled. The blood flow rate was constant at 230–250 ml/min in all patients with ESRD. Only patients with vascular access capable of maintaining a constant blood flow rate were included. All patients had the same dialysate flow rate of 500 ml/min and used the same dialyzer (FX CorDiax 60, Fresenius Medical Care, Hesse, Germany).

Before starting hemodialysis, all patients underwent the Korean version of the Montreal Cognitive Assessment (MoCA-K) test to evaluate cognitive function. This is a cognitive screening tool used to assess a person’s cognitive abilities including memory, attention, language, and visual-spatial skills^16^. Patients with ESRD who scored $\ge$ 23.5 points were defined as having normal cognitive function, whereas those with a score of $<$ 23.5 points were considered to have cognitive impairment. Laboratory tests were also conducted for all patients.

### fNIRS data acquisition

fNIRS data were acquired using a NIRSIT Lite device (OBELAB Inc., Seoul, Korea)^17^. The NIRSIT Lite is a portable, wireless, and wearable fNIRS device that measures real-time perfusion status. It measures oxyhemoglobin (HbO_2_) and deoxyhemoglobin (HbR) using the difference in the absorption rate of near-infrared light. The NIRSIT Lite has five sources and 13 detectors. It uses 15 channels to detect fNIRS signals on the prefrontal cortex^18^. The system uses near-infrared light at 780 nm and 850 nm wavelengths. The signals were measured at a sampling rate of 8.138 Hz.

For each patient, measurements were performed thrice in the resting state for 300 s while the patient was looking at a tablet PC screen with a white cross on a black background to create the same environment. The first measurement was performed 30 min before the start of hemodialysis (pre-HD), the second was 1 h after the start of hemodialysis (mid-HD), and the third was 30 min after the end of hemodialysis (post-HD).

### Data processing and creating a connectivity matrix

**Figure 1:**
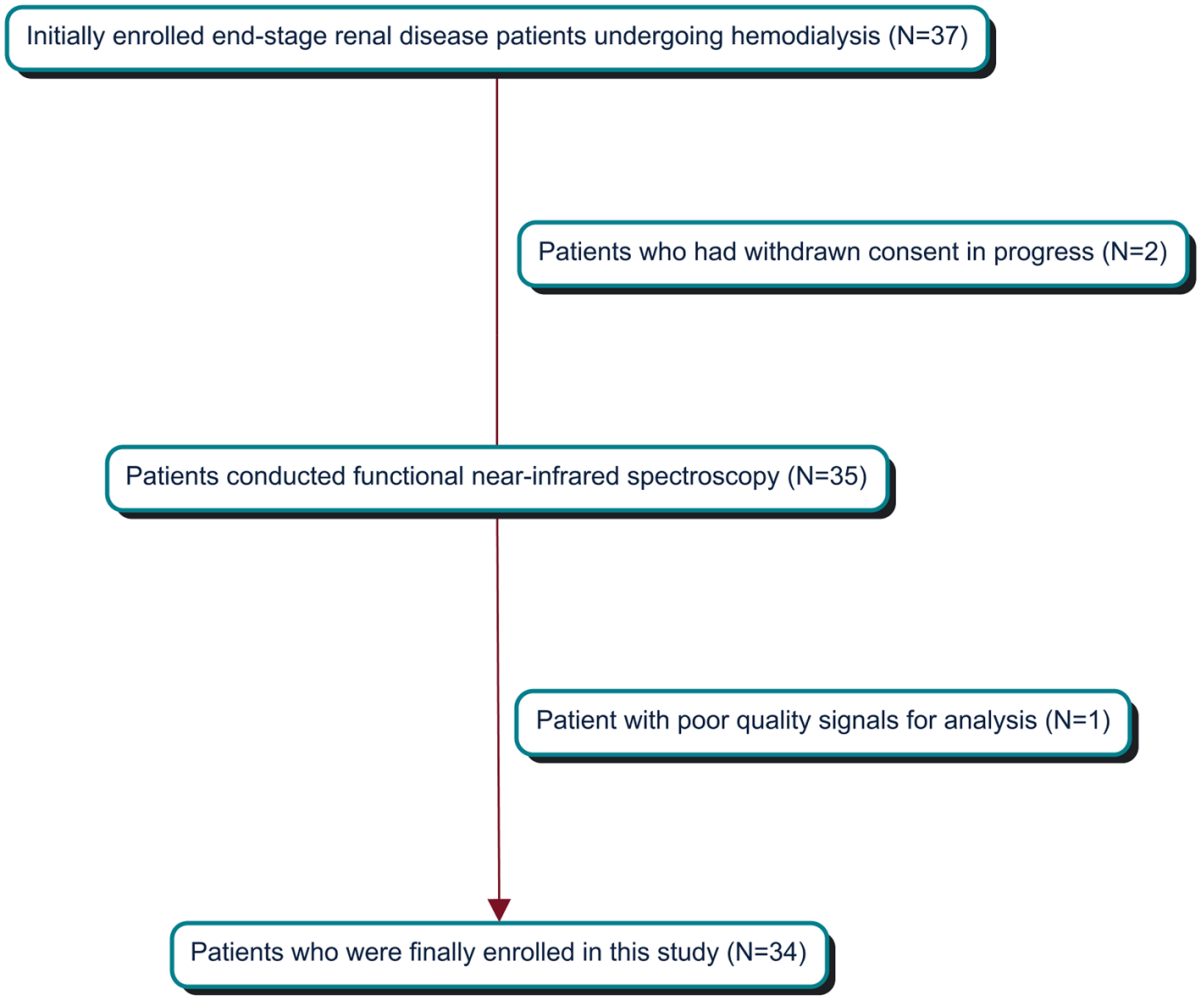
Patient selection process.

The NIRSIT Lite Analysis Tool program (version 3.2.4) was used for processing data and creating a connectivity matrix. We loaded and selected the patient data in the program. We checked the signal quality and set the signal-to-noise (SNR) ratio outliers to 2.58. This is the channel rejection criterion. If the window of a channel had an SNR value below the SNR threshold, its z-score was calculated from the SNR values of all other channels at that window point. If the average of the collected z-scores for the number of windows along a channel was larger than the outlier threshold, the channel was rejected. We excluded a patient that rejected one channel during pre-processing (Fig. 1). We applied a band-pass filter of 0.1 frequency, a low-pass cut-off value of 0.005, and a high-pass cut-off value. We processed and exported the entire data and created a weighted connectivity matrix using Pearson correlation analysis for each patient at the pre-, mid-, and post-HD periods. Three matrices were created for each patient.

### Obtaining the functional connectivity measures

Functional connectivity measures were obtained from the connectivity matrix by applying a graph theoretical analysis using BRAPH and MATLAB programs^19^. We calculated the functional connectivity measures^20,21^, including average strength, global efficiency, local efficiency, mean clustering coefficient, transitivity, and assortative coefficient at the pre-, mid-, and post-HD periods in patients with ESRD. The average strength refers to the average weighted degree of a node in a network. The global efficiency measures the effectiveness of information transfer throughout the entire network. It is the average length of the shortest inverse path between each pair of nodes in the network. The local efficiency is a measurement of the efficiency of information transfer within the network's local neighborhoods or communities. It is defined as the average length of the inverse shortest path between all pairs of nodes within a neighborhood or community. The mean clustering coefficient of a network is the average of the clustering coefficient of each node. It provides a measure of the strength of the connections between a node's neighbors. The transitivity quantifies the tendency of a network to form triangles or triplets of connected nodes. The assortative coefficient quantifies the propensity for nodes with similar degrees to be connected to one another^20,21^.

### Statistical analysis

**Figure 2:**
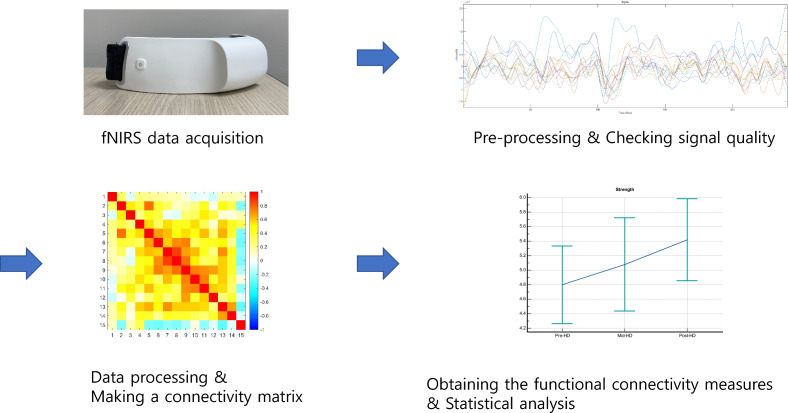
The process of analyzing functional brain connectivity in patients using functional near-infrared spectroscopy.

We compared the differences in functional connectivity measures according to hemodialysis using repeated-measures analysis of variance. Figure 2 shows the process of analyzing functional brain connectivity in patients. Bonferroni correction for multiple comparisons was applied for *p-*values and confidence intervals in the connectivity analysis, and the adjusted *p*-values were presented. In addition, a correlation analysis between clinical factors and functional connectivity measures was performed using the partial correlation method with the co-variates of age, ultrafiltration volume, and Kt/V (dialyzer clearance × time/distribution volume of urea). All statistical analyses were conducted using MedCalc® Statistical Software version 20.014 (MedCalc Software Ltd., Ostend, Belgium; https://www.medcalc.org; 2021). Statistical significance was set at *p* < 0.05.

## Results

### Patient demographic and clinical characteristics

This study included 34 patients with ESRD. Figure 1 illustrates the selection process. Table 1 shows the patient demographic and clinical characteristics. The mean age of patients was 68.5 years, and the male-to-female ratio was 2.0. The mean dialysis duration was 4.4 years. Of the 34 patients with ESRD, 23 (67.6%) had cognitive impairment, whereas 11 (32.3%) showed no cognitive impairment.

**Table 1:** Patients’ demographic and clinical characteristics.

Variables	Patients with end-stage renal disease (N = 37)
Demographic data	
Age, years	68.5 ± 12.7
Sex, male	25 (67.6)
Dialysis duration, months	53.4 ± 36.4
Years of education, years	12.3 ± 4.5
MoCA-K	20.7 ± 4.9
Body mass index	22.8 ± 4.4
Kt/V	1.6 ± 0.2
Ultrafiltration volume, ml	2032.4 ± 1081.9
Pre-HD systolic blood pressure	149.1 ± 15.6
Pre-HD diastolic blood pressure	66.7 ± 11.9
Mid-HD systolic blood pressure	143.3 ± 18.9
Mid-HD diastolic blood pressure	64.4 ± 12.5
Post-HD systolic blood pressure	148.2 ± 18.5
Post-HD diastolic blood pressure	68.7 ± 16.3
Hypotension episodes during dialysis, n	0
Comorbidities	
Hypertension	35 (94.6)
Diabetes mellitus	21 (56.8)
Laboratory data	
Hemoglobin, g/dl	10.5 ± 1.1
Iron, μg/dl	58 ± 26.6
Ferritin, ng/ml	315.6 ± 271.1
Total iron binding capacity, μg/dl	233.2 ± 39.1
Transferrin saturation, %	26.1 ± 11.7
Albumin, g/dl	4.1 ± 0.4
β2-microglobulin, mg/dl	23.5 ± 6.8
Total cholesterol, mg/dl	130.3 ± 35.7
Triglyceride, mg/dl	110.0 ± 63.9
High density lipoprotein-cholesterol, mg/dl	52.3 ± 20.4
Low density lipoprotein-cholesterol, mg/dl	56.1 ± 28.0
Calcium, mg/dl	8.6 ± 0.6
Phosphate, mg/dl	5.1 ± 2.0
Parathyroid hormone, pg/ml	274.4 ± 186.3
C-reactive protein, mg/dl	0.5 ± 1.3

Data are presented as number (%) or mean ± standard deviation.

MoCA-K: Korean version of Montreal Cognitive Assessment; Kt/V: Dialyzer clearance × time/distribution volume of urea; HD: hemodialysis.

### Differences in the functional brain connectivity according to hemodialysis

**Figure 3:**
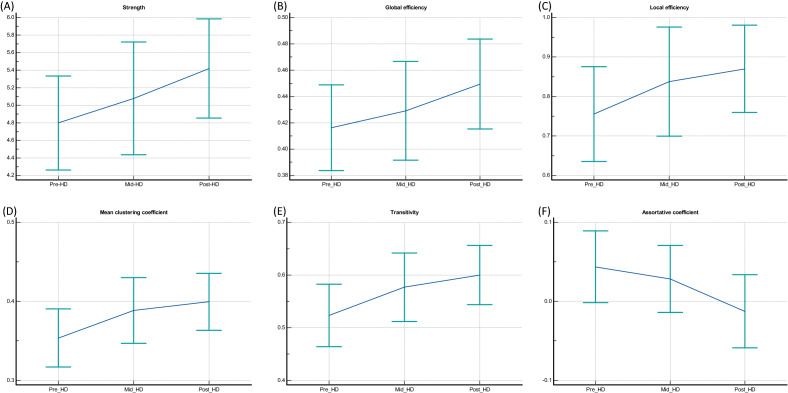
Changes in functional brain connectivity measurements in patients with ESRD based on hemodialysis. There were no differences in the average strength (**A**), global efficiency (**B**), and local efficiency (**C**) between the pre-, mid-, and post-HD periods; however, significant changes were observed in the mean clustering coefficient (**D**), transitivity (**E**), and assortative coefficient (**F**) between the pre and post-HD periods.

Table 2 and Fig. 3 show the differences in functional brain connectivity according to hemodialysis. There were significant changes in the mean clustering coefficient, transitivity, and assortative coefficient between the pre- and post-HD periods (0.353 vs. 0.399, *p* = 0.047; 0.523 vs. 0.600, *p* = 0.042; and 0.043 vs. − 0.012, *p* = 0.044, respectively). In addition, the mean clustering coefficient and transitivity values were higher in the post-HD period than in the pre-HD period. Furthermore, the assortative coefficient value was positive during the pre-HD period but negative during the post-HD period. However, there were no changes in the mean clustering coefficient, transitivity, and assortative coefficient between the pre- and mid-HD periods or between the mid- and post-HD periods. In addition, there were no significant differences in the average strength, global efficiency, and local efficiency among the pre-, mid-, and post-HD periods.

**Table 2:** Changes in the functional brain connectivity measures based on hemodialysis in patients with ESRD.

Network measures	Acquisition time	Values	Pairwise comparisons	Adjusted *p-*value	95% confidence interval
Average strength	Pre-HD	4.798	Pre-HD and Mid-HD	1.000	− 1.271 to 0.710
	Mid-HD	5.078	Pre-HD and Post-HD	0.132	− 1.370 to 0.128
	Post-HD	5.419	Mid-HD and Post-HD	0.822	− 1.113 to 0.432
Global efficiency	Pre-HD	0.416	Pre-HD and Mid-HD	1.000	− 0.070 to 0.045
	Mid-HD	0.429	Pre-HD and Post-HD	0.180	− 0.076 to 0.009
	Post-HD	0.449	Mid-HD and Post-HD	0.851	− 0.067 to 0.026
Local efficiency	Pre-HD	0.755	Pre-HD and Mid-HD	0.924	− 0.282 to 0.118
	Mid-HD	0.837	Pre-HD and Post-HD	0.174	− 0.261 to 0.032
	Post-HD	0.870	Mid-HD and Post-HD	1.000	− 0.194 to 0.129
Mean clustering coefficient	Pre-HD	0.353	Pre-HD and Mid-HD	0.442	− 0.094 to 0.024
	Mid-HD	0.388	Pre-HD and Post-HD	*0.047	− 0.091 to − 0.000
	Post-HD	0.399	Mid–-HD and Post-HD	1.000	− 0.062 to 0.040
Transitivity	Pre-HD	0.523	Pre-HD and Mid-HD	0.529	− 0.151 to 0.0441
	Mid-HD	0.576	Pre-HD and Post-HD	*0.042	− 0.152 to − 0.001
	Post-HD	0.600	Mid-HD and Post-HD	1.000	− 0.104 to 0.057
Assortative coefficient	Pre-HD	0.043	Pre-HD and Mid-HD	1.000	− 0.072 to 0.103
	Mid-HD	0.028	Pre-HD and Post-HD	*0.044	0.000 to 0.112
	Post-HD	− 0.012	Mid-HD and Post-HD	0.561	− 0.035 to 0.117

*HD* hemodialysis.

*Statistical significance.

### Association between clinical factors and functional brain connectivity

There was a significant correlation between some clinical factors and functional brain connectivity measures, including average strength and serum iron level (r = 0.355, *p* = 0.036), mean clustering coefficient and transferrin saturation (r = 0.414, *p* = 0.013), and transitivity and transferrin saturation (r = 0.395, *p* = 0.018). However, other clinical factors including age, dialysis duration, MoCA-K score, body mass index, Kt/V, ultrafiltration volume, hemoglobin, ferritin, total iron binding capacity, albumin, β2-microglobulin, total cholesterol, triglyceride, high-density lipoprotein-cholesterol, low-density lipoprotein-cholesterol, calcium, phosphate, parathyroid hormone, and C-reactive protein were not correlated with functional connectivity measures.

## Discussion

In this study, we demonstrated the feasibility of identifying functional brain connectivity using fNIRS in patients with ESRD. We also found a significant effect of hemodialysis on functional brain connectivity in these patients, showing that functional brain connectivity changed more efficiently during hemodialysis. In addition, some clinical factors were significantly correlated with functional brain connectivity measures.

We used fNIRS to investigate functional brain connectivity in patients with ESRD because it is relatively inexpensive, portable, easy to set up, and safe due to lack of radiation exposure^22^. fNIRS can be utilized for brain oxygenation monitoring and brain-computer interfaces^23^. It monitors changes in the activation energy of the cortex's superficial layers^24^. fNIRS cannot directly measure neuronal activity, but it can indirectly measure changes in brain activity by measuring the concentrations of oxygenated and deoxygenated hemoglobin in the brain's blood vessels. The changes in blood oxygenation are highly correlated with neuronal activity changes. When a particular brain region becomes active, blood flow to that region increases, resulting in an increase in oxygenated hemoglobin concentration and a decrease in deoxygenated hemoglobin concentration. Consequently, by measuring variations in the concentrations of oxygenated and deoxygenated hemoglobin, fNIRS can infer neuronal activity changes^24,25^. This means that fNIRS can be used to study the functional organization of the brain, including changes in brain activity in response to cognitive or sensory stimuli, as well as brain activity changes associated with various neuropsychiatric disorders. fNIRS is an optical technique with high temporal and spatial resolutions^15^. Moreover, it can diminish the effects of neuron geometry. It can also efficiently measure brain activity, particularly in the prefrontal cortex. Consequently, fNIRS may be valuable for evaluating brain energy absorption and neurovascular activity^15^. In this study, we demonstrated the feasibility of identifying functional brain connectivity using fNIRS in patients with ESRD. It has the advantage of continuously and repeatedly capturing images before, during, and after hemodialysis in the dialysis room. In addition, because the cognitive decline in patients with ESRD is known to be mainly associated with dysfunction of the frontal lobe, not the temporal lobe, unlike patients with Alzheimer’s disease, fNIRS is useful for evaluating brain function in these patients^26,27^. Therefore, fNIRS is considered a very suitable modality for future studies to investigate functional brain connectivity in patients with ESRD undergoing hemodialysis.

Furthermore, many rs-fMRI studies on functional brain connectivity in patients with ESRD undergoing hemodialysis have been conducted. Previous studies that used an independent component analysis algorithm reported decreased functional connectivity in the default mode network, which was associated with cognitive deficits, in patients with ESRD who underwent hemodialysis^28,29^. In addition, patients with ESRD exhibit a disruption in the topological organization of whole-brain functional networks, and an abnormal module-level interaction between the affective and cognitive control networks^7,30^. It is possible that dysfunction in brain networks contributes to the pathophysiological mechanism of cognitive impairment in patients with ESRD who undergo hemodialysis. Overall, these studies provide evidence of alterations in the intrinsic brain functional network, which may result in cognitive impairment in patients with ESRD undergoing hemodialysis.

Graph theoretical analysis can quantitatively investigate the efficacy of brain network^20,21^. In this study, we observed changes in functional brain connectivity in patients with ESRD undergoing hemodialysis using graph theory and fNIRS. Furthermore, significant changes in the mean clustering coefficient, transitivity, and assortative coefficient were observed. The mean clustering coefficient and transitivity values were higher in the post-HD period than in the pre-HD period. In graph theory, the mean clustering coefficient is defined as a factor that evaluates how well the connection between neighboring nodes around a node is^20,21^. If the mean clustering coefficient value is high, it means that the connectivity between neighboring nodes is high, suggesting that the segregation of the brain network is high. This suggests that the efficiency of information exchanges in the brain network is high. Therefore, investigating functional brain connectivity during hemodialysis is more cost-effective, and hemodialysis increases connectivity. While the difference was not statistically significant, the mean clustering coefficient gradually improved in the post-HD period than in the pre-HD period, and even in the mid-HD period when dialysis had progressed to some extent. Transitivity has a similar meaning to the mean clustering coefficient. It is the overall probability of the network having interconnected adjacent nodes, thus revealing the existence of tightly connected communities^20,21^. Therefore, the functional brain network flows in a better direction during hemodialysis. Furthermore, we found that the assortative coefficient value was positive during the pre-HD period but negative during the post-HD period. The assortative coefficient indicates whether nodes have many connections to other nodes with similar degrees or to those with very different degrees^31^. If a network has many nodes with similar degrees connected, the assortative coefficient has a positive value; otherwise, it has a negative value. A network with a positive assortative coefficient is called an assortative network, whereas a network with a negative assortative coefficient is a disassortative network^31^. Through hemodialysis, we confirmed that the assortative network changed to a disassortative one. It is well known that many technological and biological networks normally have disassortative networks that percolate less easily than assortative networks^32,33^. Therefore, it is thought that the functional brain network returns to normal in patients with ESRD undergoing hemodialysis.

Our results are consistent with those of the previous studies. Zhang et al.^34^ investigated the structural and functional alterations in the default mode network after renal transplantation in patients with ESRD and found that changes in functional connectivity recovered earlier than those in structural connectivity. Chen et al.^9^ analyzed the structural and functional networks before, and at 1 and 6 months after kidney transplantation. They showed increased functional connectivity in the default mode, dorsal attention, central executive, sensorimotor, auditory, and visual networks at 1 and 6 months after kidney transplantation. However, it remains unclear which dialyzable toxins are responsible for uremic syndrome. Small and water soluble molecules (such as urea, guanidine, phosphate, oxalate, and trimethylamine-N-oxide), lipophilic and protein binding compounds (such as p-cresyl sulfate, 3-carboxy-4-methyl-5-propyl-2-furanpropionic acid, homocysteine, indoxyl sulfate, and kynurenine), and large and medium molecules (such as beta2-microglobulin, parathyroid hormone, and advanced glycosylation end products) are considered to be the causes of uremic syndrome^35^. While some of these substances are removed by hemodialysis, the functional brain network is thought to change for the better.

Our study also demonstrated an association between functional brain connectivity measures including average strength, mean clustering coefficient, and transitivity, and some clinical factors, such as serum iron level or transferrin saturation. Iron deficiency affects the hippocampus, mitochondria, dopamine metabolism in the brain, and myelination. As a result, it is known to be linked to a deterioration in cognitive function^36^. Iron deficiency is common among patients undergoing hemodialysis due to gastrointestinal bleeding, blood collection, surgical procedures, or the dialysis treatment itself^37^. Patients undergoing hemodialysis lose an average of 1–2 g of iron annually. Adequate iron stores are required to achieve optimal hemoglobin levels. A previous study found that changes in hematocrit level were associated with altered functional connectivity in patients with ESRD^38^, and another study also discovered that decreased hemoglobin levels affected spontaneous brain activity in patients with ESRD undergoing peritoneal dialysis^39^. A possible explanation for these is that decreased hematocrit levels can result in decreased oxygen delivery to the brain, which negatively impacts brain metabolism, or that low oxygen delivery resulting from decreased hematocrit levels and altered functional connectivity may contribute to cognitive dysfunction^34^.

This study had some limitations. First, it was conducted at a single center and did not include many patients with ESRD. Second, the NIRSIT lite machine was used to obtain fNIRS data, which mainly covered the frontal lobe with only 15 channels. However, it was mainly considered that patients with ESRD had frontal lobe dysfunction, and it had the advantages of being lightweight, comfortable for scanning, and less affected by hair. Third, the patients underwent fNIRS before and after dialysis in the hemodialysis room to maintain a similar environment. Therefore, only short-term effects on functional brain connectivity after hemodialysis were evaluated; long-term effects were not determined. Although there was a statistically significant difference in functional brain connectivity after hemodialysis, the degree of the difference was not large enough to have high a *p*-value. This could have originated from short-term follow-up effects.

## Conclusion

Our study revealed the feasibility of identifying functional brain connectivity using fNIRS in patients with ESRD. We demonstrated a significant effect of hemodialysis on functional brain connectivity in these patients. Functional brain connectivity changes more efficiently during hemodialysis.

## Data Availability

The datasets used and/or analyzed during the current study available from the corresponding author on reasonable request.
